# Real-world effectiveness of DKutting Scoring Balloon for AVF and AVG stenosis and thrombosis

**DOI:** 10.1080/0886022X.2025.2553807

**Published:** 2025-09-15

**Authors:** Lihong Zhang, Fan Zhang, Xiangru Li, Jing Wen, Rui Cui, Xibin Hou, Fang Hou, Yuzhu Wang, Shen Zhan

**Affiliations:** Department of Nephrology, Haidian Hospital, Beijing, China

**Keywords:** Arteriovenous fistula, scored balloon angioplasty, hemodialysis access, vascular restenosis, real-world clinical outcomes, endovascular intervention

## Abstract

Arteriovenous fistula (AVF) and arteriovenous graft (AVG) stenosis are common complications among hemodialysis patients, necessitating repeated interventions due to neointimal hyperplasia and restenosis. Percutaneous transluminal angioplasty (PTA), while a standard treatment, faces challenges in long-term efficacy. The DKutting Scoring Balloon, a novel advancement in scoring balloon technology, incorporates triangular scoring elements and non-compliant balloon material to create controlled micro-incisions in the vessel wall, aiming to enhance procedural precision, reduce vessel trauma, and lower restenosis rates. In the largest study of its kind, this retrospective analysis conducted at Beijing Haidian Hospital evaluated the device’s performance across 428 patients with significant AVF and AVG stenosis. The findings indicate primary patency rates of 98.1%, 90.8%, and 78.0% at 1, 3, and 6 months, respectively. Subgroup analyses revealed patency rates of 79.9% for AVF and 75.4% for AVG at 6 months, demonstrating its effectiveness even in resistant and recurrent cases. Importantly, no major adverse events were reported, underscoring the device’s safety profile. These observational findings suggest that the DKutting Scoring Balloon may offer favorable outcomes compared to those typically reported with conventional PTA, though these results should be interpreted as hypothesis-generating and further randomized controlled trials are recommended to corroborate these findings and optimize its integration into clinical practice.

## Introduction

Arteriovenous fistulas (AVFs) and arteriovenous grafts (AVGs) are the two primary types of vascular access for hemodialysis in patients with end-stage renal disease (ESRD) [1,2]. Among these, AVFs are generally preferred due to their longer-term patency and fewer complications compared to alternative access methods, such as central venous catheters (CVCs). However, both AVFs and AVGs are prone to failure, predominantly due to neointimal hyperplasia, a pathological proliferation of smooth muscle cells leading to stenosis [1–3]. This stenosis reduces blood flow, necessitating frequent interventions to restore patency and ensure effective dialysis. The resulting complications significantly increase patient morbidity and mortality by heightening the risk of access thrombosis.

Percutaneous transluminal angioplasty (PTA) is the standard treatment for AV access stenosis [2,4]. Despite its widespread use, PTA has limitations, particularly a high rate of restenosis, with primary patency rates ranging between 50% and 60% at 6 months in most studies, leading to repeated interventions [2]. Traditional PTA works by applying radial pressure to dilate stenotic lesions, often resulting in uncontrolled tearing of the vessel wall, which can contribute to vessel trauma and promote restenosis [5,6]. Consequently, these drawbacks highlight the need for more precise and targeted approaches to stenosis management.

To address these challenges, scoring balloon technology has emerged as a promising alternative [7–12]. Unlike conventional balloons, scoring balloons offer the advantage of creating micro-incisions that reduce the vessel’s elastic recoil and decrease the chances of restenosis, as shown in studies using similar technologies like AngioSculpt and VascuTrak scoring balloons [11,12]. These devices employ scoring elements to create controlled incisions during inflation, effectively disrupting fibrointimal hyperplasia while minimizing vessel trauma.

The DKutting^™^ Scoring Balloon [13–15], developed by DK Medtech (Jiangsu, China), is a novel advancement in scoring balloon technology. It features three integral Nitinol coils attached to the surface of a non-compliant balloon. These coils create controlled micro-incisions in the vessel wall, aiming to reduce uncontrolled ruptures typically seen with conventional balloon angioplasty. The coils are arranged at 120° intervals to deliver uniform pressure and precise dilation during inflation. Additionally, radiopaque marker bands enhance procedural accuracy under angiography. The balloon accommodates a 0.035-inch guidewire and offers various sizes (4.0–8.0 mm in diameter, 20–80 mm in length), providing flexibility for different lesion types. A schematic illustration and photograph of the DKutting Scoring Balloon are provided in Supplemental Figure 1 to aid visual understanding of its structure, including the configuration of the Nitinol scoring coils. Its non-compliant material supports high-pressure dilation (nominal pressure of 8 atm and rated burst pressure of 20 atm), potentially improving outcomes in resistant lesions compared to traditional PTA or earlier scoring balloons.

This study presents the largest single-center analysis of scoring balloon technology to date, evaluating the real-world efficacy of the DKutting Scoring Balloon in treating AVF and AVG stenosis and thrombosis. The analysis involves a cohort of 428 patients with either AVF or AVG dysfunction, offering substantial data under consistent clinical conditions. By offering long-term follow-up data in a real-world setting, this study aims to fill a critical gap in understanding how the DKutting Scoring Balloon performs outside controlled clinical trials. The findings from this study are anticipated to significantly influence clinical practice by demonstrating the device’s potential to improve patency rates, reduce the need for repeated interventions, and offer a valuable alternative to conventional angioplasty techniques for managing dialysis access dysfunction.

## Materials and methods

### Study design

This study was a retrospective, single-center clinical evaluation conducted between 2023 and 2024 at Beijing Haidian Hospital. The primary objective was to evaluate the procedural efficacy and safety of the DKutting Scoring Balloon in the treatment of arteriovenous fistula (AVF) and arteriovenous graft (AVG) dysfunction in hemodialysis patients. All procedures were performed under fluoroscopic and ultrasound guidance. The study adhered to the Declaration of Helsinki and was approved by the Beijing Haidian Hospital Medical Ethics Committee (approval number: 2024-081). Written informed consent was obtained from all patients prior to the intervention.

### Patient selection

**Figure 1.**
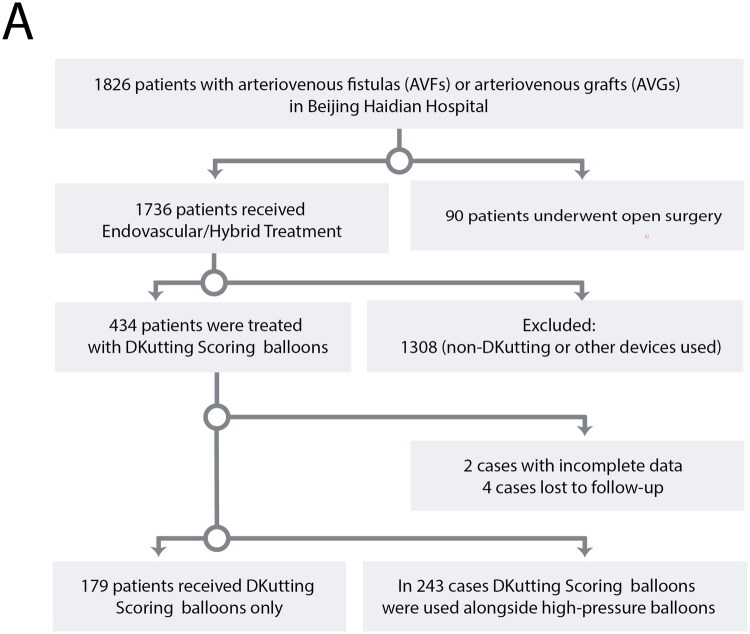
Flowchart of patient selection and treatment outcomes using the DKutting scoring balloon in AVF and AVG stenosis and thrombosis.

A total of 1,826 patients were reviewed, of whom 1,736 underwent endovascular or hybrid treatment, while 90 underwent open surgery. Among the endovascular group, 428 patients were ultimately included in this analysis. Of these, 179 (41.82%) received treatment with the DKutting Scoring Balloon alone, and 249 (58.18%) underwent combination therapy using both the DKutting Scoring Balloon and high-pressure balloons, as illustrated in the flowchart in Figure 1. This stratification was used in subsequent analyses to assess outcomes by treatment modality.

#### Inclusion criteria

Patients were included if they met the following criteria: Age ≥ 18 years; Dysfunctional or thrombosed AVF or AVG with >50% stenosis confirmed by duplex Doppler ultrasound. Willingness to provide informed consent and comply with follow-up procedures.

#### Exclusion criteria

Exclusion criteria included: Central vein or artery lesions; Use of drug-coated balloons, stents, or other scoring balloons during the study period; Life expectancy of less than 6 months.

### Procedure

For patients with stenosis, the DKutting Scoring Balloon was used according to the following procedure:

1. A balloon catheter was selected with a diameter of 1:1–1.2 times the reference vessel diameter.
2. Under ultrasound guidance, the balloon catheter was advanced along the guidewire into the target lesion.
3. The balloon was inflated at the lesion site, increasing the pressure by 1 atmosphere every 2–5 s until the wrist on the balloon disappeared, indicating successful dilation.
4. The balloon was kept inflated for 60 s, and residual stenosis was assessed. If necessary, repeat dilations were performed.
5. For multiple lesions or lesions longer than the balloon, all lesions were dilated one by one.
6. After dilation, negative pressure was applied to fully deflate and retract the balloon.
7. If residual stenosis exceeded 30% after three attempts, the procedure was considered a technical failure, and an alternative balloon or treatment was considered.

For patients with thrombosed access circuits, those with smaller thrombus volumes were treated directly with percutaneous transluminal angioplasty (PTA), while larger thrombi were manually expressed (‘milking’) before angioplasty. All procedures were performed under routine ultrasound guidance, with digital subtraction angiography (DSA) used when necessary. The balloon design, including the position of scoring coils and marker bands, can be found in Supplemental Figure 1.

In cases where DKutting balloon dilation was unsatisfactory, defined as residual stenosis >30% or a post-dilation lumen <6 mm, a larger high-pressure balloon (1–2 mm greater in diameter) was used for post-dilation. The sequence of balloon use was not systematically recorded in all patients due to limitations in the electronic medical records. However, in standard practice, the scoring balloon was used first for controlled incision, followed by high-pressure dilation only when needed.

### Endpoints

The primary efficacy endpoint was target lesion primary patency [16] (defined as freedom from reintervention) at 1, 3, and 6 months post-procedure. Technical success was defined as residual stenosis of <30%, and clinical success as the resumption of normal dialysis for at least one session following the intervention. The secondary endpoint was cumulative access circuit patency at 6 months, a measure encompassing the interval from the study procedure to access abandonment. Notably, cumulative access circuit patency included cases where multiple interventions were necessary to maintain secondary patency, reflecting instances of reocclusion requiring additional interventions or open surgery.

### Data collection and analysis

Demographic, clinical, and procedural data were collected using standardized forms. These included: patient characteristics (age, biologic sex, primary disease, comorbidities); access characteristics (type of access, fistula age, number of prior interventions, lesion location, stenosis length); procedural details (balloon diameter, number of devices used). Follow-up was conducted regularly with both physical examinations and ultrasound imaging. Additional symptom-driven follow-ups were conducted as needed.

### Statistical analysis

Patient characteristics, lesion information, and balloon utilization were reported for both the AVG and AVF groups. The Shapiro-Wilk test was employed to assess the normality of continuous data. Normally distributed data were expressed as mean ± standard deviation (x̅ ± s), while non-normally distributed data were presented as medians with interquartile ranges [M (Q1, Q3)]. Categorical variables were reported as frequencies and percentages. Between-group comparisons (AVG vs. AVF) for continuous data were conducted using independent sample *t*-tests or the Mann-Whitney U test, while categorical variables were compared using the chi-square test or Fisher’s exact test.

Primary patency rates (frequency and percentage) at months 1, 3, and 6 were reported for the aggregate population, as well as separately for AVG and AVF. Survival analyses were conducted using the Kaplan-Meier method, and the log-rank test was employed to compare survival distributions between the two groups. Additionally, patency rates were reported according to various pre-intervention procedures, stent stenosis, thrombosis, and different lesion types. The chi-squared test was applied to assess differences among patients who underwent various pre-intervention procedures.

Statistical analyses were conducted using SAS 9.4 and the R programming language (version 4.3.3) for data processing and visualization. Statistical significance was determined using a two-sided p-value of less than 0.05.

To address concerns about baseline differences and potential confounding, we used a multivariable Cox proportional hazards regression model (Supplemental Table 1). This analysis adjusted for key variables, including patient demographics (age, sex), dialysis age, access type and age (AVF vs. AVG), comorbidities (diabetes mellitus, cardiovascular and cerebrovascular disease), lesion site (e.g., inflow artery, anastomosis, outflow vein, puncture area, or other), number of prior interventions, and balloon type (scoring balloon alone vs. combined high-pressure use). These variables were selected based on clinical relevance and known influence on patency outcomes. Results from this analysis support the robustness of our primary findings and confirm that the observed differences are unlikely to be solely due to selection bias.

## Results

### Demographic and lesion characteristics

In this retrospective study at Beijing Haidian Hospital (2023–2024), we analyzed 428 patients, including 248 with AVF and 180 with AVG, all presenting with significant stenosis or thrombosis leading to access dysfunction. The mean age was similar between groups (AVF: 60.87 years; AVG: 60.57 years). Notably, AVG patients had a longer median dialysis duration (57 vs. 36 months, *p* < 0.001) and a higher proportion of females (61.11% vs. 48.39%, *p* = 0.009).

Chronic glomerulonephritis was the most common underlying condition, more prevalent among AVF patients (78.23% vs. 68.33%). Comorbidities such as hypertension were widespread (88.08%), but diabetes and coronary heart disease were significantly higher in the AVG group (diabetes: 47.78% vs. 36.69%, *p* = 0.022; coronary heart disease: 33.89% vs. 22.18%, *p* = 0.007).

AVF patients frequently had radial artery-cephalic vein fistulas (94.76%), whereas brachial artery-basilic vein grafts were predominant in the AVG group (57.22%). Lesion site analysis revealed the puncture segment was the predominant site in both AVF (92.34) and AVG cases (72.78). Detailed statistics are summarized in Tables 1 and 2.

**Table 1.** Demographics and comorbidities among AVF and AVG patients.

	Total (*n* = 428)	AVG (*n* = 180)	AVF (*n* = 248)	Statistic	*p*
Age, mean ± SD	60.74 ± 11.51	60.57 ± 11.56	60.87 ± 11.50	*T* = −0.27	0.79
Dialysis age (months), M (Q₁, Q₃)	44.50 (18.00, 90.50)	57.00 (29.00, 103.25)	36.00 (12.00, 84.00)	*Z* = −3.76	<0.001
Fistula age (months), M (Q₁, Q₃)	33.00 (12.00, 60.00)	33.50 (15.00, 58.25)	33.00 (12.00, 60.25)	*Z* = −0.34	0.733
Sex, *n* (%)				χ² = 6.79	0.009
Female	230 (53.74)	110 (61.11)	120 (48.39)		
Male	198 (46.26)	70 (38.89)	128 (51.61)		
Primary disease, *n* (%)				χ² = 0.385	0.535
Other diseases	23 (5.37)	10 (5.56)	13 (5.24)		
Polycystic kidney disease	25 (5.84)	12 (6.67)	13 (5.24)		
Chronic glomerulonephritis	317 (74.07)	123 (68.33)	194 (78.23)		
Diabetes	58 (13.55)	34 (18.89)	24 (9.68)		
Hypertension	5 (1.17)	1 (0.56)	4 (1.61)		
Hypertension, *n* (%)				χ² = 1.81	0.179
None	51 (11.92)	17 (9.44)	34 (13.71)		
Have	377 (88.08)	163 (90.56)	214 (86.29)		
Diabetes, *n* (%)				χ² = 5.28	0.022
None	251 (58.64)	94 (52.22)	157 (63.31)		
Have	177 (41.36)	86 (47.78)	91 (36.69)		
Coronary heart disease, *n* (%)				χ² = 7.24	0.007
None	312 (72.90)	119 (66.11)	193 (77.82)		
Have	116 (27.10)	61 (33.89)	55 (22.18)		
Cerebrovascular disease, *n* (%)				χ² = 0.09	0.762
None	392 (91.59)	164 (91.11)	228 (91.94)		
Have	36 (8.41)	16 (8.89)	20 (8.06)		

*p* Values were generated using independent sample *t*-test, Mann-Whitney U test, chi-square test or Fisher’s exact test.

**Table 2.** Lesion site distribution in AVF and AVG patients, *n* (%).

	AVG (*n* = 180)	AVF (*n* = 248)
Lesion site		
Arterial inflow	35 (19.44)	35 (14.11)
Anastomosis	100 (55.56)	114 (45.97)
Outflow vein	99 (55.00)	45 (18.15)
Puncture segment vein	131 (72.78)	229 (92.34)
other	6 (3.33)	1 (0.40)
Cephalic vein	2 (1.11)	0 (0.00)
Reflux vein stent inside/edge	4 (2.22)	1 (0.40)
Arterial inflow-venous outflow		
Brachial artery-cephalic vein	34 (18.89)	6 (2.42)
Brachial artery-brachial vein	15 (8.33)	0 (0.00)
Brachial artery-basilic vein	103 (57.22)	0 (0.00)
Brachial artery-median cubital vein	27 (15.00)	4 (1.61)
Brachial artery-internal jugular vein	1 (0.56)	0 (0.00)
Radial artery-cephalic vein	0 (0.00)	235 (94.76)
Ulnar artery-basilic vein	0 (0.00)	3 (1.21)

### Primary patency rates

This study evaluated 428 patients, comprising 248 with arteriovenous fistulas (AVFs) and 180 with arteriovenous grafts (AVGs), all treated using the DKutting Scoring Balloon. As summarized in Table 3, most patients required only one or two balloons, with 75% of AVF and 83% of AVG patients falling into this category. Most patients presented one or two stenotic lesions: 44.76% of AVF and 26.11% of AVG patients had a single lesion. Among the various balloon sizes, the 7 mm DKutting balloon was the most frequently used, particularly in the AVG group, where it was employed in 75% of cases.

**Table 3.** Balloon utilization, lesion count, and pretreatment in AVF vs. AVG groups, *n* (%).

	Total (*n* = 428)	AVG (*n* = 180)	AVF (*n* = 248)
Number of balloons used, *n* (%)			
1	179 (41.82)	91 (50.56)	88 (35.48)
2	159 (37.15)	59 (32.78)	100 (40.32)
3	69 (16.12)	23 (12.78)	46 (18.55)
4	19 (4.44)	7 (3.89)	12 (4.84)
5	2 (0.47)	0 (0.00)	2 (0.81)
Number of lesions, *n* (%)			
1	158 (36.92)	47 (26.11)	111 (44.76)
2	190 (44.39)	90 (50.00)	100 (40.32)
3	62 (14.49)	28 (15.56)	34 (13.71)
4	17 (3.97)	14 (7.78)	3 (1.21)
5	1 (0.23)	1 (0.56)	0 (0.00)
Dingke balloon size (mm), *n* (%)			
4	24 (5.61)	0 (0.00)	24 (9.68)
5	34 (7.94)	9 (5.00)	25 (10.08)
6	110 (25.70)	36 (20.00)	74 (29.84)
7	260 (60.75)	135 (75.00)	125 (50.40)
Number of other balloon treatments before this intervention, *n* (%)			
0	146 (34.11)	34 (18.89)	112 (45.16)
1	86 (20.09)	30 (16.67)	56 (22.58)
2	56 (13.08)	26 (14.44)	30 (12.10)
3	41 (9.58)	19 (10.56)	22 (8.87)
4	26 (6.07)	15 (8.33)	11 (4.44)
≥5	73 (17.06)	56 (31.11)	17 (6.85)

At 6 months, both groups demonstrated high primary patency rates (Table 4). The AVF group achieved an overall patency of 79.92%. Notably, patients without prior interventions reached a patency rate of 82.57%, while those with five or more prior interventions had a slightly reduced rate of 70.59%. In the AVG group, the overall patency was higher at 75.43%, with an higher 87.04% patency rate in patients who had undergone five or more interventions.

**Table 4.** Primary patency outcomes at 6 months for AVF and AVG with various Pre-intervention procedures, *n* (%).

Primary patency rate, *n* (%)	Total	0	1	2	3	4	≥5	*p*
Month 1	419/427(98.13)	143/146(97.95)	84/86(97.67)	53/55(96.36)	41/41(100.00)	25/26(96.15)	73/73(100.00)	0.484
Month 3	383/422(90.76)	125/144(86.81)	81/85(95.29)	47/55(85.45)	37/41(90.24)	25/26(96.15)	68/71(95.77)	0.086
Month 6	327/419(78.04)	112/143(78.32)	71/84(84.52)	33/54(61.11)	32/41(78.05)	20/26(76.92)	59/71(83.10)	0.032
AVG								
Month 1	177/180(98.33)	33/34(97.06)	29/30(96.67)	26/26(100.00)	19/19(100.00)	14/15(93.33)	56/56(100.00)	0.257
Month 3	162/177(91.53)	26/34(76.47)	27/29(93.10)	25/26(96.15)	17/19(89.47)	14/15(93.33)	53/54(98.15)	0.018
Month 6	132/175(75.43)	22/34(64.71)	22/28(78.57)	15/25(60.00)	14/19(73.68)	12/15(80.00)	47/54(87.04)	0.088
AVF								
Month 1	242/247(97.98)	110/112(98.21)	55/56(98.21)	27/29(93.10)	22/22(100.00)	11/11(100.00)	17/17(100.00)	0.577
Month 3	221/245(90.20)	99/110(90.00)	54/56(96.43)	22/29(75.86)	20/22(90.91)	11/11(100.00)	15/17(88.24)	0.076
Month 6	195/244(79.92)	90/109(82.57)	49/56(87.50)	18/29(62.07)	18/22(81.82)	8/11(72.73)	12/17(70.59)	0.084

*p* Values were generated using Fisher’s exact test.

**Figure 2.**
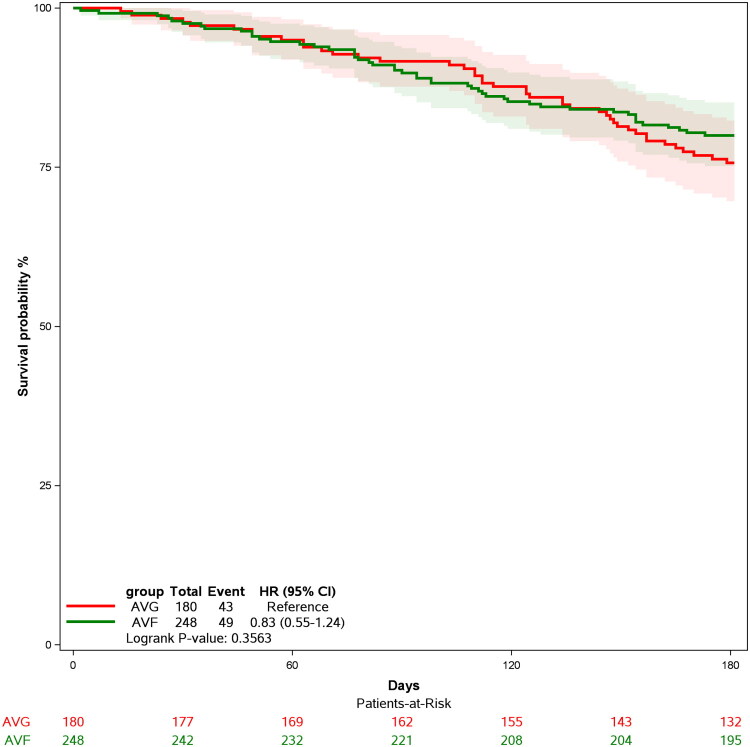
Kaplan-Meier survival curves for AVF and AVG groups.

These outcomes are illustrated in the Kaplan-Meier survival analysis (Figure 2), which tracks primary patency over 180 days. The survival probabilities for both AVF and AVG groups were comparable, with a hazard ratio of 0.83 (95% CI: 0.55–1.24) for AVG compared to AVF. Statistical analysis using the log-rank test revealed no significant difference between the two groups (*p* = 0.3563).

These findings underscore the DKutting Scoring Balloon’s consistent efficacy across diverse patient profiles, including those with multiple prior interventions. The comparable survival probabilities observed in the Kaplan-Meier analysis further supports its reliability in maintaining primary patency. Given these high patency rates, the device appears effective for both AVF and AVG populations, regardless of prior intervention history.

### Multivariable analysis of factors affecting primary patency

To account for potential confounding variables, a multivariable Cox proportional hazards regression model was conducted to evaluate predictors of primary patency at 6 months (Supplemental Table 1). Covariates included age, sex, dialysis age, AVF or AVG age, presence of diabetes, cardiovascular or cerebrovascular disease, lesion site, number of prior interventions, and treatment strategy (scoring balloon alone vs. combined therapy). The analysis showed that male sex and fistula age was independently associated with better primary patency. Use of the scoring balloon alone was not significantly inferior to combination therapy. These findings suggest that even after adjusting for clinically relevant baseline characteristics, the DKutting Scoring Balloon demonstrates consistent efficacy. However, the unexpectedly high patency in high-risk AVG patients with ≥5 prior interventions may reflect underlying selection bias or unmeasured procedural factors.

### Patency outcomes in subgroups with stent stenosis, thrombosis and different lesion types

We further analyzed patency outcomes in AVG patients with and without stent stenosis (Supplemental Table 2). While patients with stent stenosis experienced marginal, non-significant reductions in patency rates in months 3 and 6 (*p* > 0.05), overall rates remained high. A detailed breakdown by stenotic and coexistence of thrombosis revealed that the DKutting Scoring Balloon consistently maintained primary patency rates above 73.53% at 6 months (Supplemental Table 3), outperforming standard benchmarks.

These results underscore the device’s robustness, even in patients with complex clinical profiles requiring multiple prior interventions. The DKutting Scoring Balloon’s advanced scoring mechanism enhances lumen expansion, contributing to its exceptional performance and utility in diverse patient populations.

### Effectiveness of single-balloon treatment and outcomes in high-risk patients

**Figure 3.**
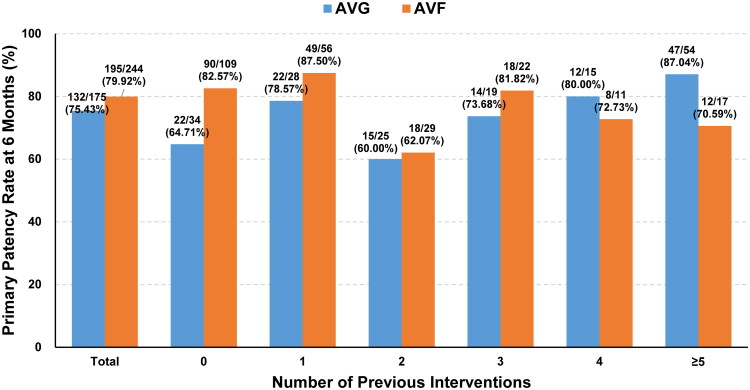
Primary patency at 6 months for AVF and AVG patients by number of pre-intervention procedures.

In evaluating the DKutting Scoring Balloon as a stand-alone intervention, our analysis focused on patients who received single-balloon treatment, excluding those who underwent combination therapy with high-pressure balloons. This approach allowed a clearer assessment of the balloon’s independent efficacy. Our findings highlight the efficacy of single-balloon treatments in maintaining vascular access patency among high-risk patients, as detailed in Table 5. In the AVF group, the 6-month primary patency rate was 80.23%, compared to an impressive 80.68% in the AVG group. Notably, Figure 3 shows that AVF patients with no prior interventions achieved an 82.57% patency rate, whereas those with ≥5 interventions experienced a reduction to 70.59%. Conversely, AVG patients with ≥5 prior interventions exhibited a remarkable 87.04% patency rate, highlighting the balloon’s efficacy in complex cases.

**Table 5.** Primary patency outcomes at 6 months with DKutting scoring balloon (one balloon per case), *n* (%).

	Month	Overall	Stenosis	Thrombosis
Overall				
	1	175/178 (98.31)	167/170 (98.24)	8/8 (100.00)
	3	165/176 (93.75)	158/168 (94.05)	7/8 (87.50)
	6	140/174 (80.46)	134/166 (80.72)	6/8 (75.00)
AVF				
	1	85/87 (97.70)	84/86 (97.67)	1/1 (100.00)
	3	81/87 (93.10)	80/86 (93.02)	1/1 (100.00)
	6	69/86 (80.23)	68/85 (80.00)	1/1 (100.00)
AVG				
	1	90/91 (98.90)	83/84 (98.81)	7/7 (100.00)
	3	84/89 (94.38)	78/82 (95.12)	6/7 (85.71)
	6	71/88 (80.68)	66/81 (81.48)	5/7 (71.43)

Table 6 provides insights into the characteristics of AVG patients with multiple prior interventions. A median graft age of 5 years, combined with a high prevalence of chronic glomerulonephritis (69.64%), likely influenced these outcomes. Additionally, structural changes in the grafts, such as small pseudoaneurysm in the puncture segment, appear to enhance patency by simulating the favorable hemodynamics typically associated with AVFs.

**Table 6.** Demographic and clinical characteristics of patients with ≥5 pre-intervention procedures.

	Total (*n* = 73)	AVF (*n* = 17)	AVG (*n* = 56)
Age; M (Q₁, Q₃)	62 (56, 67)	59 (56, 65)	63 (56, 68)
Dialysis age (month); M (Q₁, Q₃)	96 (70, 132)	84 (66, 132)	102.5 (71, 132)
Fistula age (month); M (Q₁, Q₃)	65 (46, 82)	79 (47, 89)	60.5 (44.5, 75)
Sex; *n* (%)			
Female	51 (69.86)	11 (64.71)	40 (71.43)
Male	22 (30.14)	6 (35.29)	16 (28.57)
Primary renal disease; *n* (%)			
Other disease	5 (6.85)	2 (11.76)	3 (5.36)
Polycystic kidney	7 (9.59)	3 (17.65)	4 (7.14)
Chronic glomerulonephritis	49 (67.12)	10 (58.82)	39 (69.64)
Diabetes	11 (15.07)	1 (5.88)	10 (17.86)
Hypertension	1 (1.37)	1 (5.88)	–
Hypertension; *n* (%)	63 (86.3)	14 (82.35)	49 (87.5)
Diabetes; *n* (%)	29 (39.73)	3 (17.65)	26 (46.43)
Coronary heart disease; *n* (%)	26 (35.62)	4 (23.53)	22 (39.29)
Cerebrovascular disease; *n* (%)	8 (10.96)	1 (5.88)	7 (12.5)

These findings support the DKutting Scoring Balloon as a vital tool in both restoring and maintaining vascular access, particularly in patients with resistant lesions or extensive procedural histories. This innovation has the potential to significantly improve clinical outcomes in hemodialysis care, offering a robust solution to a long-standing challenge.

### Comparative outcomes: single vs. combination therapy

To assess the specific contribution of the DKutting Scoring Balloon, patients were stratified into two cohorts: those receiving single-balloon therapy (DKutting only) and those undergoing combination therapy (DKutting plus high-pressure balloon). This stratification aimed to isolate the balloon’s effect by minimizing potential confounding from adjunctive interventions.

As shown in Table 5 and Figure 3, patients treated exclusively with the DKutting Scoring Balloon achieved robust 6-month primary patency rates, 80.23% in the AVF group and 80.68% in the AVG group. These outcomes closely mirrored those of the overall cohort, underscoring the balloon’s independent efficacy.

Furthermore, multivariable Cox regression analysis (Supplemental Table 1) demonstrated no statistically significant difference in patency risk between single-balloon and combination therapy groups. This suggests that the addition of a high-pressure balloon did not yield a measurable benefit over the DKutting Scoring Balloon alone in this clinical setting.

### Balloon utilization and clinical efficacy

Analysis of balloon size utilization, as detailed in Table 7, revealed significant differences across stenosis and thrombosis subgroups. In AVF stenosis, the 7 mm balloon predominated (53.64%), while AVF thrombosis showed a more varied distribution, favoring 6 mm (35.71%) and 7 mm (25.00%). Similarly, AVG stenosis cases overwhelmingly utilized 7 mm balloons (85.00%), whereas AVG thrombosis relied on 6 mm (52.50%) and 7 mm (40.00%) sizes. The chi-square test confirmed significant variability in balloon size distribution for AVF (*p* = 0.008) and AVG groups (*p* < 0.001). Our data indicate that 7 mm balloons are favored for stenotic conditions across both AVF and AVG cases, potentially reflecting a better efficacy profile. In contrast, smaller balloons (6 mm) are more commonly employed in thrombosis cases, likely due to anatomical or procedural considerations. These results highlight the role of balloon size in optimizing treatment outcomes for different subgroups.

**Table 7.** Distribution of scored balloon sizes across stenosis and thrombosis subgroups in AVF and AVG patients.

Group	Stenosis (*n* = 220)	Thrombosis (*n* = 28)	χ²	*p*
AVF group			–	0.008
4 mm	19 (8.64)	5 (17.86)		
5 mm	19 (8.64)	6 (21.43)		
6 mm	64 (29.09)	10 (35.71)		
7 mm	118 (53.64)	7 (25.00)		
AVG Group			36.2	<0.001
4 mm	0	0		
5 mm	6 (4.29)	3 (7.50)		
6 mm	15 (10.71)	21 (52.50)		
7 mm	119 (85.00)	16 (40.00)		

*p* Values were generated using chi-square test.

## Discussion

Arteriovenous access (AVF and AVG) dysfunction, primarily caused by stenosis, remains a significant complication in hemodialysis patients [1–3]. Traditional balloon angioplasty has been widely used to restore arteriovenous access patency, but challenges such as restenosis and resistant lesions have limited its efficacy [7–9]. In recent years, the introduction of drug-coated balloons and stents for managing stenoses or thrombi in access reflux veins has shown promising results. These devices have demonstrated enhanced outcomes by potentially reducing restenosis rates and improving long-term patency [17–24]. While scoring balloon technologies have shown promise, drug-coated balloons (DCBs) and stent grafts are established alternatives for managing AV access stenosis, particularly in resistant or recurrent lesions. Multiple randomized controlled trials, such as those by Lookstein et al. and Yin et al., have demonstrated the efficacy of paclitaxel-coated balloons in prolonging primary patency compared to standard PTA [17,18]. However, concerns remain regarding their cost, potential for downstream embolization, and recent safety controversies surrounding paclitaxel exposure in peripheral arteries. Stent grafts, such as those evaluated by Haskal et al. and Vesely et al., offer superior patency in select settings like venous anastomosis stenosis, but at the expense of higher procedural complexity and the risk of edge restenosis or stent migration [22,23]. In this context, the DKutting Scoring Balloon presents an attractive middle-ground: offering mechanical precision without the need for pharmacologic agents or permanent implants. This study provides a detailed assessment of the DKutting Scoring Balloon’s efficacy, comparing the results to previous studies that used different scoring and high-pressure balloon technologies.

Several important observations from this study emerge when viewed alongside previous research. For instance, Holden et al. reported a technical success rate of 100% using the Cook Advance Enforcer 35 Focal-Force PTA Balloon Catheter, yet primary patency rates were only 62.0% at 3 months and 25.1% at 6 months [10]. In contrast, our study shows significantly higher patency rates (Table 4), indicating a stronger overall performance of the DKutting Scoring Balloon. The DKutting Scoring Balloon features three integral Nitinol coils affixed to the surface of a non-compliant balloon. These coils create controlled micro-incisions in the vessel wall, potentially reducing the uncontrolled ruptures often seen with conventional balloon angioplasty. The coils are arranged at 120° intervals to provide focused and uniform pressure during inflation, ensuring precise dilation of narrowed vessels. The low patency rates in Holden’s study were attributed to several factors, including the treatment of long-segment lesions and the limited burst pressure of the balloon, which ranged from 10 to 16 atm. Furthermore, active imaging follow-up in that study may have contributed to increased detection of restenosis, leading to more frequent reinterventions and impacting patency outcomes.

In comparison, Sun et al.’s study on the BD VascuTrak balloon demonstrated higher patency rates, especially for AVF dysfunction resistant to conventional angioplasty [11]. Their results showed a primary patency rate of 88.2% at 6 months and 74.5% at 12 months, underscoring the potential of scoring balloon technologies in treating challenging cases. Additionally, the DKutting Scoring Balloon is compatible with a standard 0.035-inch guidewire and offers various balloon diameters (4.0–8.0 mm) and lengths (20–80 mm), providing flexibility to address different lesion types. This configuration aims to produce deeper, controlled micro-incisions in the vessel thickening while potentially reducing random tearing. Our study demonstrates encouraging patency rates at 6 months with the DKutting Scoring Balloon, which may reflect a potential advantage in selected scenarios (Figure 2; Table 4). However, due to the retrospective, non-comparative design of our study, these findings should be considered exploratory and require validation in randomized controlled trials. Another comparative study reported patency rates of 90% at 2 months and 80% at 6 months for a mixed cohort of AVF and AVG patients [12]. These studies illustrate the variability in outcomes based on lesion characteristics and follow-up methods.

In our cohort of 428 patients, which included a substantial percentage of restenosis cases (34.11%), the DKutting Scoring Balloon achieved outstanding primary patency rates of 98.1%, 90.8%, and 78.0% at 1, 3, and 6 months, respectively (Table 4; Supplemental Table 3). These results represent some of the highest patency rates reported when compared with other balloon angioplasty technologies. Notably, the subgroup analysis of AVF patients showed a 6-month primary patency rate of 79.92% (Table 4; Figure 3), which is comparable to or better than other studies. Importantly, patients who had undergone multiple prior interventions also maintained high patency rates, reinforcing the device’s effectiveness in treating recurrent stenosis. In the AVG group, the 6-month primary patency rate was even higher at 75.43%, with some patients who had undergone five or more previous interventions achieving a remarkable 87.04% patency rate (Tables 4 and 6). Further analysis may be needed to explain this phenomenon, possibly linked to altered hemodynamics in older fistulas.

Our findings revealed that larger balloons were associated with slightly higher patency rates, particularly in AVG cases, where the hemodynamic profile may allow for more aggressive dilation strategies. For AVF cases, medium-sized balloons provided optimal outcomes, balancing effective dilation with minimal trauma. These findings align with the notion that lesion-specific balloon selection could further optimize outcomes. Notably, the procedural success rate remained high across all balloon sizes, supporting the versatility of the DKutting Scoring Balloon in managing diverse lesion profiles.

Several factors contribute to the exceptional outcomes observed with the DKutting Scoring Balloon. The device’s triangular nickel-titanium coil springs enable deeper incisions into the vessel wall, especially in cases of fibrointimal hyperplasia. Its non-compliant material, with a nominal pressure of 8 atmospheres and a rated burst pressure of 20 atmospheres, promises stable, high-pressure dilation without overexpansion. The cutting depth of 0.36 mm allows for more effective treatment of resistant lesions. The balloon’s high burst pressure of 20 atm, coupled with its non-compliant design, provides the necessary radial force to dilate stenotic segments uniformly, reducing the risk of irregular tearing that can contribute to restenosis [7–9]. The directional scoring system of the DKutting balloon, with its fixed scoring elements, minimizes vascular injury during retraction, which could explain the high success and low complication rates in this study.

In this study, more than half of the cases (58.18%) required multiple balloon inflations, usually at the same lesion site. Our institutional protocol typically began with a DKutting Scoring Balloon sized 1:1 to the reference vessel. In approximately 5–10% of cases where this approach yielded insufficient dilation (residual stenosis >30% or luminal diameter <6 mm), a larger high-pressure balloon (1–2 mm wider) was used subsequently. This stepwise escalation was designed to enhance acute lumen gain while minimizing vessel trauma. Balloon size selection was individualized based on vessel dimensions and lesion resistance, with high-pressure balloons reserved for cases of inadequate response.

Although procedural details such as inflation pressure and sequence frequency were not uniformly recorded due to the retrospective nature of the study, this is acknowledged as a limitation. To address this and other data gaps, a prospective, randomized controlled trial is currently in development. It will systematically evaluate procedural sequences, balloon pressure protocols, and diameter selection, as well as incorporate standardized measurements of thrombus characteristics (e.g., segment length, volume) and detailed anatomical data to better assess their influence on patency outcomes.

Despite promising results, the retrospective design carries inherent limitations. Inconsistent documentation of thrombosed segment length prevented its inclusion in the analysis. Given the known impact of thrombus burden on procedural success and long-term patency, this omission introduces unmeasured variability, especially in subgroup analyses of thrombosed cases. These variables will be specifically addressed in the forthcoming prospective trial to provide more granular insight into treatment efficacy.

Another limitation stems from the symptom-driven follow-up used in this study, which diverges from imaging-based surveillance protocols employed in trials such as those by Lookstein et al. [17] and Dolmatch et al. [24]. While symptom-driven monitoring reflects real-world practice, it may delay the detection of subclinical restenosis and thus overestimate patency rates. This methodological difference complicates cross-study comparisons and may partly explain the favorable outcomes observed with the DKutting Scoring Balloon in our cohort.

The exclusion of patients with severe hyperplasia near the anastomosis could also introduce selection bias, as these patients are typically associated with worse outcomes. Moreover, in cases where residual stenosis was <30% but luminal diameter remained <6 mm, additional post-dilation with high-pressure balloons was performed. This likely improved acute lumen expansion and may have positively influenced overall patency rates (Table 5). Future analysis will aim to isolate the effect of this adjunctive strategy.

To mitigate confounding, a multivariable Cox regression analysis (Supplemental Table 1) was conducted, adjusting for both patient- and lesion-level characteristics. Lesion location and comorbidities such as diabetes were identified as independent predictors of patency. However, the unexpectedly high patency observed in AVG patients with ≥5 prior interventions may reflect selection bias, given their older graft age (median: 65 months) (Table 6) and susceptibility to pseudoaneurysm-related dilation benefits. These subgroup findings warrant further prospective validation.

Additional limitations include potential selection biases in subgroup analyses and unmeasured confounders such as operator technique or lesion calcification. Incomplete documentation of thrombus volume and balloon pressure, combined with the small sample sizes in specific subgroups (e.g., stent-edge stenosis, AVG thrombosis), limits generalizability. These issues will be systematically addressed in the upcoming trial, which is designed to capture comprehensive procedural and anatomical variables.

Subgroup comparisons showed similar patency outcomes between patients treated with the DKutting Scoring Balloon alone and those receiving combination therapy with high-pressure balloons. Although multivariate modeling (Table 5; Supplemental Table 1) adjusted for this factor and found no significant difference, the retrospective nature of the study precludes definitive conclusions. Randomized trials will be needed to determine if combination therapy offers incremental benefits.

No major adverse events were observed. Minor complications, including small hematomas and localized vessel ruptures (e.g., endothelial tears from the scoring wire), were conservatively managed without progression to flow-limiting dissection or surgical intervention. Operator experience likely contributed to the low clinical impact. However, due to the retrospective design, minor or transient events may have been underreported. Comprehensive safety monitoring will be a core component of the planned prospective study to fully characterize the risk profile of the DKutting Scoring Balloon.

## Conclusions

In summary, the DKutting Scoring Balloon offers a highly effective option for treating AVF and AVG dysfunction, with promising patency rates and an acceptable safety profile in this real-world setting. Nevertheless, prospective randomized trials are necessary to confirm these preliminary observations and establish comparative effectiveness. Future randomized controlled trials will be critical to confirm these findings and further investigate the device’s long-term efficacy and safety in diverse patient populations.

## Supplementary Material

Supplemental Material

Supplemental Material

Supplemental Material

Supplemental Material

## Data Availability

The datasets generated during and/or analyzed during the current study are available from the corresponding author on reasonable request.
