# Supplementary material for: Real-world effectiveness of DKutting Scoring Balloon for AVF and AVG stenosis and thrombosis
Source: Ren Fail. 2025 Sep 15;47(1):2553807. doi: 10.1080/0886022X.2025.2553807 (PMC12444924; doi:10.1080/0886022X.2025.2553807)
Supplement: Supplemental Material [file IRNF_A_2553807_SM5279.docx]

**Supplemental Table 3, Comparison of Primary Patency Rates Across Access Types and Lesion Characteristics at 1, 3, and 6 Months**

| Access type | Month 1 | Month 3 | Month 6 |
| --- | --- | --- | --- |
| Overall | 419/427(98.13) | 383/422(90.76) | 327/419(78.04) |
| Stenosis | 352/359(98.05) | 323/354(91.24) | 277/351(78.92) |
| Thrombosis | 67/68(98.53) | 60/68(88.24) | 50/68(73.53) |
| AVF | 242/247(97.98) | 221/245(90.20) | 195/244(79.92) |
| Stenosis | 214/219(97.72) | 196/217(90.32) | 172/216(79.63) |
| Thrombosis | 28/28(100.00) | 25/28(89.29) | 23/28(82.14) |
| AVG | 177/180(98.33) | 162/177(91.53) | 132/175(75.43) |
| Stenosis | 138/140(98.57) | 127/137(92.70) | 105/135(77.78) |
| Thrombosis | 39/40(97.50) | 35/40(87.50) | 27/40(67.50) |
