# Supplementary material for: Real-world effectiveness of DKutting Scoring Balloon for AVF and AVG stenosis and thrombosis
Source: Ren Fail. 2025 Sep 15;47(1):2553807. doi: 10.1080/0886022X.2025.2553807 (PMC12444924; doi:10.1080/0886022X.2025.2553807)
Supplement: Supplemental Material [file IRNF_A_2553807_SM5278.docx]

**Supplemental Table 2. Comparison of Patency Rates in the AVG Group with and without Stent Stenosis**

| **Primary patency rate , n(%)** | **Total (n = 180)** | **None (n = 176)** | **Yes (n = 4)** | ***P*** |
| --- | --- | --- | --- | --- |
| Month 1 | 177/180(98.33) | 173/176(98.30) | 4/4(100.00) | . |
| Month 3 | 162/177(91.53) | 159/173(91.91) | 3/4(75.00) | . |
| Month 6 | 132/175(75.43) | 130/171(76.02) | 2/4(50.00) | . |

P values were generated using Fisher’s exact test.
