# Supplementary material for: Real-world effectiveness of DKutting Scoring Balloon for AVF and AVG stenosis and thrombosis
Source: Ren Fail. 2025 Sep 15;47(1):2553807. doi: 10.1080/0886022X.2025.2553807 (PMC12444924; doi:10.1080/0886022X.2025.2553807)
Supplement: Supplemental Material [file IRNF_A_2553807_SM5276.docx]

|  |  | **Univariate Cox model** | | **Multivariate Cox model** | | **Stepwise multivariate Cox model** | |
| --- | --- | --- | --- | --- | --- | --- | --- |
| **Population** | **Risk factor** | **HR (95% CI)** | **P value** | **HR (95% CI)** | **P value** | **HR (95% CI)** | **P value** |
| **Overall** | Group: Autologous | Ref . |  | Ref . |  | Ref . |  |
|  | Artificial | 1.21(0.8,1.83) | 0.357 | 1.18(0.67,2.07) | 0.574 |  | . |
|  | Gender: Female | Ref . |  | Ref . |  | Ref . |  |
|  | Male | 0.64(0.42,0.98) | 0.042 | 0.62(0.4,0.97) | 0.037 | 0.64(0.42,0.98) | 0.042 |
|  | Age | 1.01(0.99,1.02) | 0.492 | 1(0.98,1.02) | 0.704 |  | . |
|  | Dialysis age | 1(0.99,1) | 0.213 | 1(0.99,1) | 0.628 |  | . |
|  | Fistula age | 1(0.99,1) | 0.133 | 1(0.99,1) | 0.391 |  | . |
|  | Primary disease diabetes | 0.84(0.45,1.57) | 0.583 | 0.72(0.36,1.44) | 0.35 |  | . |
|  | Hypertension | 1.75(0.81,3.79) | 0.153 | 1.53(0.69,3.38) | 0.295 |  | . |
|  | Diabetes | 1.15(0.76,1.74) | 0.498 | 1.16(0.71,1.89) | 0.563 |  | . |
|  | Coronary heart disease | 1.08(0.69,1.7) | 0.733 | 1.04(0.63,1.73) | 0.873 |  | . |
|  | Cerebrovascular disease | 1.18(0.59,2.34) | 0.64 | 1.18(0.58,2.43) | 0.645 |  | . |
|  | Arterial anastomosis/arterial inflow | 0.98(0.56,1.7) | 0.942 | 0.86(0.47,1.56) | 0.616 |  | . |
|  | Venous anastomosis/anastomosis | 1.04(0.69,1.56) | 0.854 | 0.92(0.6,1.43) | 0.722 |  | . |
|  | Outflow vein/puncture segment Vein | 0.95(0.59,1.54) | 0.837 | 1.02(0.58,1.8) | 0.951 |  | . |
|  | AVG puncture area/outflow vein | 1.14(0.76,1.72) | 0.527 | 1.13(0.65,1.96) | 0.671 |  | . |
|  | Number of balloons used : > 1 | Ref . |  | Ref . |  | Ref . |  |
|  | 1 | 0.79(0.52,1.2) | 0.271 | 0.71(0.45,1.12) | 0.138 |  | . |
|  | Number of other balloon Treatments before surgery : >0 | Ref . |  | Ref . |  | Ref . |  |
|  | 0 | 1.03(0.67,1.58) | 0.907 | 1.1(0.68,1.8) | 0.696 |  | . |
| **AVG** | Gender: Female | Ref . |  | Ref . |  | Ref . |  |
|  | Male | 0.94(0.51,1.74) | 0.844 | 0.74(0.39,1.43) | 0.375 |  | . |
|  | Age | 1(0.97,1.02) | 0.776 | 1(0.97,1.03) | 0.871 |  | . |
|  | Dialysis age | 1(0.99,1) | 0.207 | 1(0.99,1.01) | 0.667 |  | . |
|  | Fistula age | 0.98(0.97,1) | 0.011 | 0.99(0.97,1.01) | 0.18 | 0.98(0.97,1) | 0.011 |
|  | Primary disease diabetes | 1.15(0.55,2.4) | 0.703 | 0.96(0.41,2.25) | 0.919 |  | . |
|  | Hypertension | 1.04(0.37,2.91) | 0.94 | 1.32(0.43,4.07) | 0.63 |  | . |
|  | Diabetes | 1.42(0.78,2.6) | 0.249 | 2(0.93,4.31) | 0.075 |  | . |
|  | Coronary heart disease | 0.73(0.38,1.43) | 0.36 | 0.7(0.33,1.48) | 0.35 |  | . |
|  | Cerebrovascular disease | 0.68(0.21,2.19) | 0.517 | 0.57(0.16,2.04) | 0.387 |  | . |
|  | Arterial anastomosis/arterial inflow | 0.88(0.41,1.89) | 0.738 | 1.29(0.53,3.15) | 0.572 |  | . |
|  | Venous anastomosis/anastomosis | 0.73(0.4,1.32) | 0.298 | 0.62(0.32,1.24) | 0.177 |  | . |
|  | Outflow vein/puncture segment Vein | 1.42(0.77,2.64) | 0.262 | 1.16(0.58,2.33) | 0.682 |  | . |
|  | AVG puncture area/outflow vein | 0.53(0.28,0.98) | 0.043 | 0.59(0.28,1.26) | 0.174 |  | . |
|  | Number of balloons used : > 1 | Ref . |  | Ref . |  | Ref . |  |
|  | 1 | 0.59(0.32,1.1) | 0.096 | 0.65(0.33,1.27) | 0.211 |  | . |
|  | Number of other balloon Treatments before surgery : >0 | Ref . |  | Ref . |  | Ref . |  |
|  | 0 | 1.96(1.01,3.82) | 0.048 | 1.47(0.62,3.53) | 0.383 |  | . |
| **AVF** | Gender: Female | Ref . |  | Ref . |  | Ref . |  |
|  | Male | 0.49(0.27,0.89) | 0.018 | 0.49(0.26,0.94) | 0.032 |  | . |
|  | Age | 1.01(0.99,1.04) | 0.244 | 1.01(0.98,1.04) | 0.531 |  | . |
|  | Dialysis age | 1(0.99,1) | 0.449 | 1(0.99,1) | 0.384 |  | . |
|  | Fistula age | 1(0.99,1.01) | 0.823 | 1(0.99,1.01) | 0.945 |  | . |
|  | Primary disease diabetes | 0.37(0.09,1.51) | 0.164 | 0.31(0.07,1.44) | 0.135 |  | . |
|  | Hypertension | 2.64(0.82,8.48) | 0.103 | 2.27(0.68,7.63) | 0.185 |  | . |
|  | Diabetes | 0.92(0.51,1.65) | 0.769 | 1.09(0.53,2.23) | 0.823 |  | . |
|  | Coronary heart disease | 1.48(0.8,2.76) | 0.211 | 1.56(0.78,3.11) | 0.209 |  | . |
|  | Cerebrovascular disease | 1.75(0.74,4.11) | 0.2 | 2.46(0.94,6.46) | 0.067 |  | . |
|  | Arterial anastomosis/arterial inflow | 1.06(0.47,2.35) | 0.894 | 0.98(0.4,2.38) | 0.964 |  | . |
|  | Venous anastomosis/anastomosis | 1.36(0.77,2.38) | 0.287 | 1.38(0.76,2.51) | 0.288 |  | . |
|  | Outflow vein/puncture segment Vein | 0.5(0.21,1.17) | 0.109 | 0.64(0.23,1.76) | 0.386 |  | . |
|  | AVG puncture area/outflow vein | 2.06(1.11,3.83) | 0.023 | 2.02(0.98,4.19) | 0.058 |  | . |
|  | Number of balloons used : > 1 | Ref . |  | Ref . |  | Ref . |  |
|  | 1 | 0.95(0.53,1.72) | 0.871 | 1.01(0.52,1.96) | 0.972 |  | . |
|  | Number of other balloon Treatments before surgery : >0 | Ref . |  | Ref . |  | Ref . |  |
|  | 0 | 0.77(0.43,1.37) | 0.377 | 0.82(0.44,1.52) | 0.534 |  | . |

Univariate, multivariate, and stepwise multivariate Cox regression models were used to assess associations between clinical and procedural variables and 6-month primary patency loss. Hazard ratios (HR) >1 indicate increased risk of patency loss. Variables with “Ref.” represent the reference group against which the HR of the comparison category was calculated. Separate analyses were performed for the overall population, AVG subgroup, and AVF subgroup. Bolded values and asterisks (*) denote statistical significance (p < 0.05).
