# Supplementary figures and images for: Real-world effectiveness of DKutting Scoring Balloon for AVF and AVG stenosis and thrombosis
Source: Ren Fail. 2025 Sep 15;47(1):2553807. doi: 10.1080/0886022X.2025.2553807 (PMC12444924; doi:10.1080/0886022X.2025.2553807)

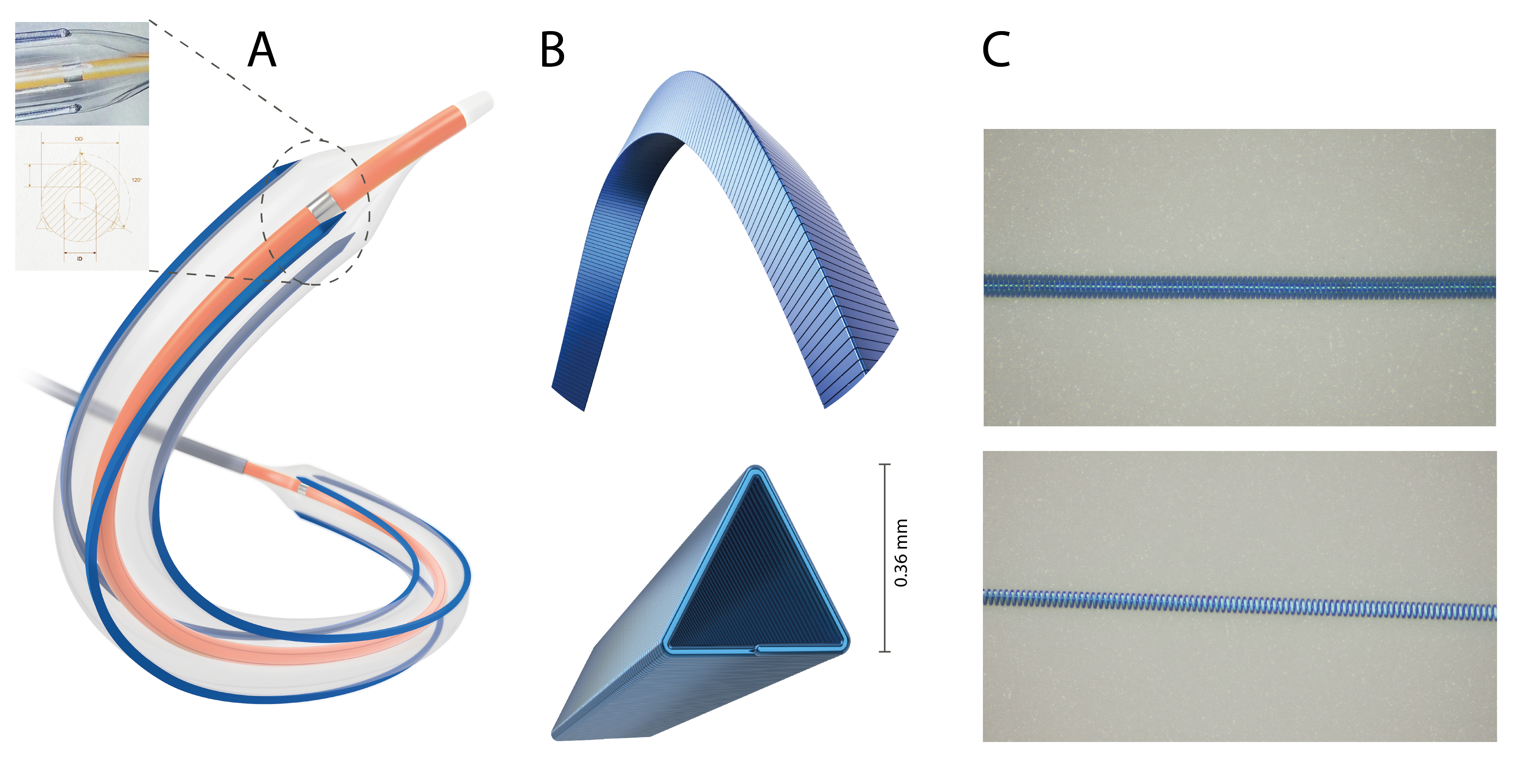

Supplement: Supplemental Material [file IRNF_A_2553807_SM5273.png]
